# Surveillance of Physicians Causing Potential Drug-Drug Interactions in Ambulatory Care: A Pilot Study in Switzerland

**DOI:** 10.1371/journal.pone.0147606

**Published:** 2016-01-25

**Authors:** Heiner C. Bucher, Rita Achermann, Nadja Stohler, Christoph R. Meier

**Affiliations:** 1 Basel Institute for Clinical Epidemiology & Biostatistics, Department of Clinical Research, University Hospital Basel, Basel, Switzerland; 2 Institute for Clinical Pharmacy and Epidemiology, Department of Pharmaceutical Sciences, University of Basel, Basel, Switzerland; 3 Hospital Pharmacy, University Hospital Basel, Basel, Switzerland; Penn State College of Medicine, UNITED STATES

## Abstract

**Objectives:**

We analysed potential drug-drug interactions (DDI) in ambulatory care in Switzerland based on claims data from three large health insurers in 2010 to identify physicians with peculiar prescription behaviour differing from peers of the same specialty.

**Methods:**

We analysed contraindicated or potentially contraindicated DDI from the national drug formulary and calculated for each physician the ratios of the number of patients with a potential DDI divided by the number of patients at risk and used a zero inflated binomial distribution to correct for the inflated number of observations with no DDI. We then calculated the probability that the number of caused potential DDI of physicians was unlikely (p-value < 0.05 and ≥0.01) and very unlikely (p-value <0.01) to be due to chance.

**Results:**

Of 1'607'233 females and 1'525'307 males 1.3% and 1.2% were exposed to at least one potential DDI during 12 months. When analysing the 40 most common DDI, 598 and 416 of 18,297 physicians (3.3% and 2.3%) were causing potential DDI in a frequency unlikely (p<0.05 and p≥0.01) and very unlikely (p<0.01) to be explained by chance. Patients cared by general practitioners and cardiologists had the lowest probability (0.20 and 0.26) for not being exposed to DDI.

**Conclusions:**

Contraindicated or potentially contraindicated DDI are frequent in ambulatory care in Switzerland, with a small proportion of physicians causing potential DDI in a frequency that is very unlikely to be explained by chance when compared to peers of the same specialty.

## Background

Potential drug-drug interactions (DDI) are a common drug-related problem in ambulatory care and may lead to adverse drug reactions with serious consequences [1]. In epidemiological surveys, between 0.1% and 2.6% of hospitalisations are related to adverse drug reactions from DDI [2, 3]. Rates for elderly patients are higher, ranging from 0.7% to 6.4% [2]. Potential DDI may be inevitable in patients with comorbidities in need of multiple drugs, and the risk of adverse clinical consequences may be reduced by appropriate dose adjustment and monitoring. Many potential DDI may not be clinically relevant or can be handled if monitored adequately; some DDI, however, can be deleterious, and concomitant use of such drugs has to be strongly discouraged. For example, co-administration of rifampicin, a bactericidal antibiotic against tuberculosis with potent cytochrome P450 enzyme induction properties, should not be combined with anti-HIV protease inhibitors or with new oral anticoagulants, as plasma levels of the latter drugs may substantially decrease and lead to antiretroviral therapy failure with serious consequences for the patient. Potential DDI are an explicit indicator for the evaluation of the appropriateness of medication in ambulatory care and for measuring physician performance [4]. Explicit indicators such as DDI can be applied to large data, but generally cannot address other factors such as comorbidities which likely influence drug prescribing decision leading to DDI. Quantifying potential DDI may not necessarily be suitable as marker for the quality of therapy of physicians for particular patients; clinical judgement and implicit criteria are needed to achieve this detailing. However, this approach is time consuming, depends on users’ knowledge and attitude, and may lack reliability [5].

In Switzerland no population-based data on the prevalence of potential DDI in ambulatory care exist, and no indicators for the quality of ambulatory care are routinely used. The goal of this pilot study was to investigate the frequency of DDI in the Swiss population using health insurer claims data and to explore whether individual physicians causing potential DDI at higher frequency than their peers of the same specialty can be identified. In theory this would open the possibility to establish feedback or benchmark systems for physicians with peculiar drug prescription behaviour to better understand and to potentially improve their prescription behaviour.

## Methods

We assessed the frequency of potential DDI in patients of all age using claim data from three large health insurance companies in Switzerland in 2010. Reimbursement claims from pharmacists and self-dispensing physicians and hospital outpatients are electronically processed by these insurers. The system provides information on the date the prescription drug was issued, the delivery date, the active ingredient, the drug formulation, the amount of the active ingredient, and the number of dispensed units of a drug. In the reimbursement system prescribed drugs can be identified by a specific ‘pharmacode’ (www.e-mediat.ch), and prescribing physicians by a unique registration number (‘Zahlstellenregisternummer’). For the purpose of this study, the provided data files did not contain information potentially allowing the identification of individual patients. According to Swiss law ethical approval for routinely gathered claim data is not mandatory if data files are entirely anonymized.

For the reporting of all potential DDI we included drug combinations of single compounds or drug classes prescribed by physicians or outpatient clinics classified as contraindicated (grade 1) or potentially contraindicated (grade 2) based on the Pharmavista database version 2011. For the comparison of physicians we restricted the number of contraindicated and potentially contraindicated drug combinations to 40. We excluded all DDI we encountered in our database with less than 50 patients for contraindicated and less than 250 patients for potentially contraindicated drugs. We also disregarded all clinically not relevant DDI (own judgement) and DDI that can be avoided with appropriately scheduled drug intake. Contraceptives are not reimbursed by Swiss health insurers and therefore are not recorded. The Pharmavista database is tailored to the Swiss market and allows for the identification of DDI with the use of ATC codes from the Anatomical Therapeutic Chemical Classification system, an internationally accepted classification system for drug consumption studies provided by WHO (http://www.whocc.no). The database covers 86% of drug interaction information provided by Stockley’s Drug Interaction compendium [6]. A list of these DDI combinations is provided in the S1 Table.

To identify potential DDI the overlapping drug use periods had to be calculated. We first determined the treatment duration of each drug because this information is not contained in the claims database. We made an assumption that the number of days of intake is on average equal to the number of defined daily doses (DDD) per active substance prescribed. In case of a subsequent prescription of the same active substance, the next prescription date was taken to determine when an intake period ended; otherwise, the length of the intake period was set to the number of DDD. The information on DDD was taken from Galdat, a database provided by e-mediat (e-mediat AG Schönbühl); in case of missing information for DDD we consulted a database provided by WHO (http://www.whocc.no/atc_ddd_index/). For the determination of all overlapping drug intake time periods, we assumed that patients were actually taking both drugs for the entire period. Differences between the estimated and actually drug intake period can obviously lead to a biased determination of potential DDI. Such differences are more likely to occur if a patient takes two or more drugs for shorter periods than if one drug has to be taken on a regular basis (e.g. oral anticoagulant in a patient with atrial fibrillation), with a second drug with a potential DDI being added. Therefore we checked the lag time periods between delivery dates of potentially interacting drugs.

Patients may have multiple exposures to the same potential DDI caused by one or more physicians caring for the same patient. In this project we aimed to identify the physician responsible for a potential DDI by issuing a prescription on top of another pre-existing treatment. The prescription date does not always reflect the date when a physician had contact with a patient. Some prescriptions can be renewed by the pharmacists without seeing the physician, but these were included in the analyses as well. For each prescription we assigned the preceding consultation date with the physician who issued the prescription. If a conflicting drug was already prescribed at this time point, the physician was considered responsible for the potential DDI. If a physician caused in the same patient multiple potential DDI with the same drugs, we counted only the first potential DDI. If the same DDI in the same patient was caused by a second physician, this was counted as a new potential DDI and assigned to the second physician.

The likelihood for potential DDI depends on the patient population under study. Physicians caring for patients with multiple comorbidities are more likely to prescribe several drugs and to cause potential DDI. We lacked comorbidity parameters to adjust for patient case mix; we therefore restricted for each physician the patient collective to those patients who were at risk for a potential DDI. We defined being at risk as taking at least one of the two drugs of a DDI from our predefined list. In case of a DDI, typically one drug is usually taken over a longer period and the other only for a short period. Therefore we considered only patients at risk who were chronically exposed to a long-term treatment with a drug. For example, to explore the potential DDI for statins and macrolide antibiotics, only patients having received statins were included in the patient collective at risk. The drugs used to identify the patient collective at risk are marked in S1 Table (drug class A and B). Because the clinical relevance of the relatively prevalent DDI of clopidogrel and proton pump inhibitors is controversially discussed we present our result with the inclusion and exclusion of this DDI.[7]

For each physician we calculated the ratios (*r*_*P*_*)* of the number of patients with a potential DDI (*n*_*P*_) divided by the number of patients at risk (*Ω*_*P*_*)*. Thus, we formed for each physician a reference population, that allowed to calculate the proportion of patients with DDI at the physician level. Our model is based on the assumption that populations at risk for drug interactions (*Ω*_*P*_) taken care by a given physician are comparable. Given the variety of ratios for DDI across specialities we decided to compare DDI only within specialties. The distribution of the ratios *r*_*P*_ was modelled as a zero inflated binomial distribution (with the two coefficients π_zero,_ π_bin_) to correct for the inflated number of observations with zero DDI in our dataset. The underlying assumption is that with probability π_zero_, the ratio *r*_*P*_ is 0, and with probability 1 – π_zero_, a binomial random variable (π_bin_) is observed. Thus, the coefficients π_zero_, corrects for the overrepresentation of physicians with no patients exposed to DDI and is expressed as probability between 0 and 1. The higher the coefficient (π_zero_) the higher is the probability that zero patients of physicians from a given specialty have been exposed to a potential DDI. The coefficient π_bin_ represents the proportions of patients per physician exposed to potential DDI if the outcome variable *r*_*P*_ follows with probability 1 – π_zero_ a binomial distribution.

For each physician, we calculated the probability of being at the upper tail of the zero inflated binomial distribution. If the probability was <0.05 and ≥0.01 we classified the prescription behaviour of a physician to cause a potential DDI as ‘unlikely to be due to chance’, and if the probability was <0.01 we regarded the prescription behaviour to cause a potential DDI as ‘very unlikely to be due to chance’.

In a pre-processing step, we imported data from the insurance companies into a MySQL database. All analyses were carried out using R version 3.0.1 (R Foundation for Statistical Computing, Vienna, Austria). The parameters for the zero inflated binomial distribution were estimated with the R-library Vector Generalized Linear and Additive Models VGAM [8].

## Results

The analysis was based on 282,780,644 claim data entries and 32,817,874 prescriptions from three major health insurers covering 3,132,540 individuals (40% of the Swiss population) with known Swiss residence in 2010. Of claims processed by these insurers 5.4% contained insufficient information of prescribed drugs and therefore could not be included. Among 20,710 registered physicians 19,630 prescribed at least one drug. The median number of patients per physician in the study population was 290, [Interquartile range (IQR) 23 to 418]. Sixty percent of all drugs were prescribed by 7845 general practitioners to 1’567’000 patients; of these, 48% of prescribed drugs were self-dispensed by the doctor without involving a pharmacy. In the study population the number of individuals aged 70 years and older was overrepresented (14.7%) when compared with the general Swiss population (11.9% aged 70 years or older), as were individuals from the French speaking part of Switzerland (16.4% study population, 18.8% Swiss population).

Of 1'607'233 females and 1'525'307 males 1.3% of females and 1.2% of males received a drug combination which is contraindicated or potentially contraindicated, and 0.4% of females and 0.5% of males received a drug combination which is contraindicated (Table 1). The rate for exposure to at least one contraindicated drug combination was slightly higher for females than for males, and for individuals aged 70 or older of both sexes (Table 1).

**Table 1:** Number of insured individuals with contraindicated or potentially contraindicated drug-drug interactions (DDI) in the year 2010.

	Swiss population		Study population			Individuals with contraindicated and potentially contraindicated DDI			Individuals with contraindicated DDI					
Age group	Females	Males	Females	%	Males	%	Females	%	Males	%	Females	%	Males	%	
0–49	2'466'341	2'531'821	925'074	37.5	947'535	37.4	4498	0.5	3‘177	0.3	769	0.1	828	0.1
50–69	952'944	940'516	405'146	42.5	393'519	41.8	6661	1.6	7‘171	1.8	2492	0.6	3154	0.8
70+	554'597	378'691	277'013	49.9	184'253	48.7	9774	3.5	7‘668	4.2	3120	1.1	2831	1.5
Total	3'973'882	3'851'028	1'607'233	40.4	1'525'307	39.6	20'933	1.3	18‘016	1.2	6‘381	0.4	6‘813	0.5

All contraindicated and potentially contraindicated DDI prescribed by physicians in ambulatory care are included.

Based on our probability definitions, 416 of 18,297 (2.3%) physicians prescribed drugs leading to potential DDI in a frequency that is very unlikely (p<0.01) to be explained by chance and 598 physicians (3.3%) prescribed drugs leading to potential DDI in a frequency unlikely (p<0.05 p≥0.01) to be explained by chance (Table 2). The probability to cause DDI differed substantially between specialties. For example the coefficient to account for the probability of no DDI (π_zero_) from the binominal distribution was high for ophthalmologist (0.72) indicating that a high proportion of ophthalmologists did not cause any DDI whereas the coefficient in general practitioners (0.20) indicated that the proportion of general practitioners causing no DDI was substantially lower. General practitioners (0.20), cardiologist (0.26), physicians in group practices (0.30) and oncologists (0.37) showed the lowest coefficient of π_zero_ indicating that in these specialities there were the lowest number of patients with no DDI. When we excluded DDI by clopidogrel and proton pump inhibitors the coefficient increased in particular for cardiologist (0.34) indicating that a considerable proportion of DDIs caused by these specialists is due to this drug combination.

**Table 2:** Number of physicians by speciality with patient exposed to drug-drug interactions (DDI) in the year 2010 unlikely (p<0.05) and very unlikely (p<0.01) to be explained by chance*.

Specialty	Physicians with ≥1 patient at risk for DDIn	Patients with DDI n (%)	π_zero_ (Probability of no DDI)**	π_bin_ (Probability of DDI)***	Physicians causing DDI unlikely to be by chance (p<0.05 & p≥0.01) n (%)	Physicians causing DDI very unlikely to be by chance (p<0.01) n (%)	Patients with DDIn (%)	π_zero_ (Probability of no DDI)**	π_bin_ (Probability of DDI)***	Physicians causing DDI unlikely to be by chance (p<0.05 & p≥0.01) n (%)	Physicians causing DDI very unlikely to be by chance (p<0.01) n (%)
Drug-drug interactions including clopidogrel-proton pump inhibitors							Drug-drug interactions without clopidogrel proton pump inhibitors				
Cardiologist	*395*	999 (3.1)	0.26	0.02	13 (3.3)	20 (5.1)	639 (2.4)	0.34	0.01	15 (3.8)	16 (4.1)
Dermatologist	*389*	389 (1.2)	0.50	0.01	14 (3.6)	8 (2.1)	388 (1.5)	0.50	0.01	13 (3.3)	8 (2.1)
General practioner	*7247*	23,650 (74.1)	0.20	0.03	312(4.3)	216 (3.0)	19,400 (73.0)	0.23	0.02	289 (4.0)	182 (2.5)
Gynecologist	*1149*	400 (1.3)	0.73	0.01	18(1.6)	7 (0.6)	396 (1.5)	0.73	0.01	20 (1.7)	6 (0.5)
Oncologist	*166*	238 (0.8)	0.37	0.02	7 (4.2)	4 (2.4)	202 (0.8)	0.43	0.02	9 (5.4)	4 (2.4)
Ophthalmologist	*1062*	501 (1.6)	0.72	0.01	38 (3.6)	36 (3.4)	465 (1.8)	0.73	0.01	36 (3.4)	33 (3.1)
Other	*3504*	2803 (8.9)	0.66	0.02	72 (2.1)	65 (1.9)	2356 (8.9)	0.69	0.02	71 (2.0	47 (1.3)
Pediatrician	*941*	375 (1.2)	0.71	0.01	25 (2.7)	9 (1.0)	373 (1.4)	0.71	0.01	23 (2.4)	11(1.2)
Group practice	*121*	504 (1.6)	0.30	0.02	2 (1.7)	6 (5.0)	430 (1.6)	0.30	0.01	4 (3.3)	5 (4.1)
Psychiatrist	*2953*	1482 (4.6)	0.63	0.02	86 (2.9)	31 (1.1)	1469 (5.5)	0.63	0.02	84 (2.8)	31 (1.0)
Rheumatologist	*370*	575 (1.8)	0.40	0.02	11 (3.0)	14 (3.8)	450 (1.7)	0.47	0.01	10 (2.7)	8 (2.2)
Total	18,297	31,916			598 (3.3)	416 (2.3)	26,568			574 (3.1)	351 (1.9)

*Patients can be exposed at multiple DDI

** Coefficient π_zero_, corrects for the overrepresentation of physicians with no patients exposed to DDI and is expressed as probability between 0 and 1. The higher the coefficient the higher is the probability that no patients of physicians from a given specialty have been exposed to a potential DDI.

***The cofficient π_bin_ represents the proportions of patients within a specialty exposed to potential DDI and is expressed as P_bin_ = 1-pzero for the zero inflated binomial distribution. Numbers are provided with and without the potential DDI clopidogrel—proton pump inhibitors

The probabilities for DDI were highest for general practitioners (π_bin_ 0.03), oncologists (π_bin_ 0.02), other physicians (π_bin_ 0.02), and psychiatrists (π_bin_ 0.02). Although the coefficients π_bin_ for general practitioners and oncologists are not considerably different, the coefficients of π_zero_ for these two disciplines indicate that probability of patients cared by an oncologist not to be exposed to a DDI is about 50% higher than of patients served by a general practitioner.

**Fig 1:**
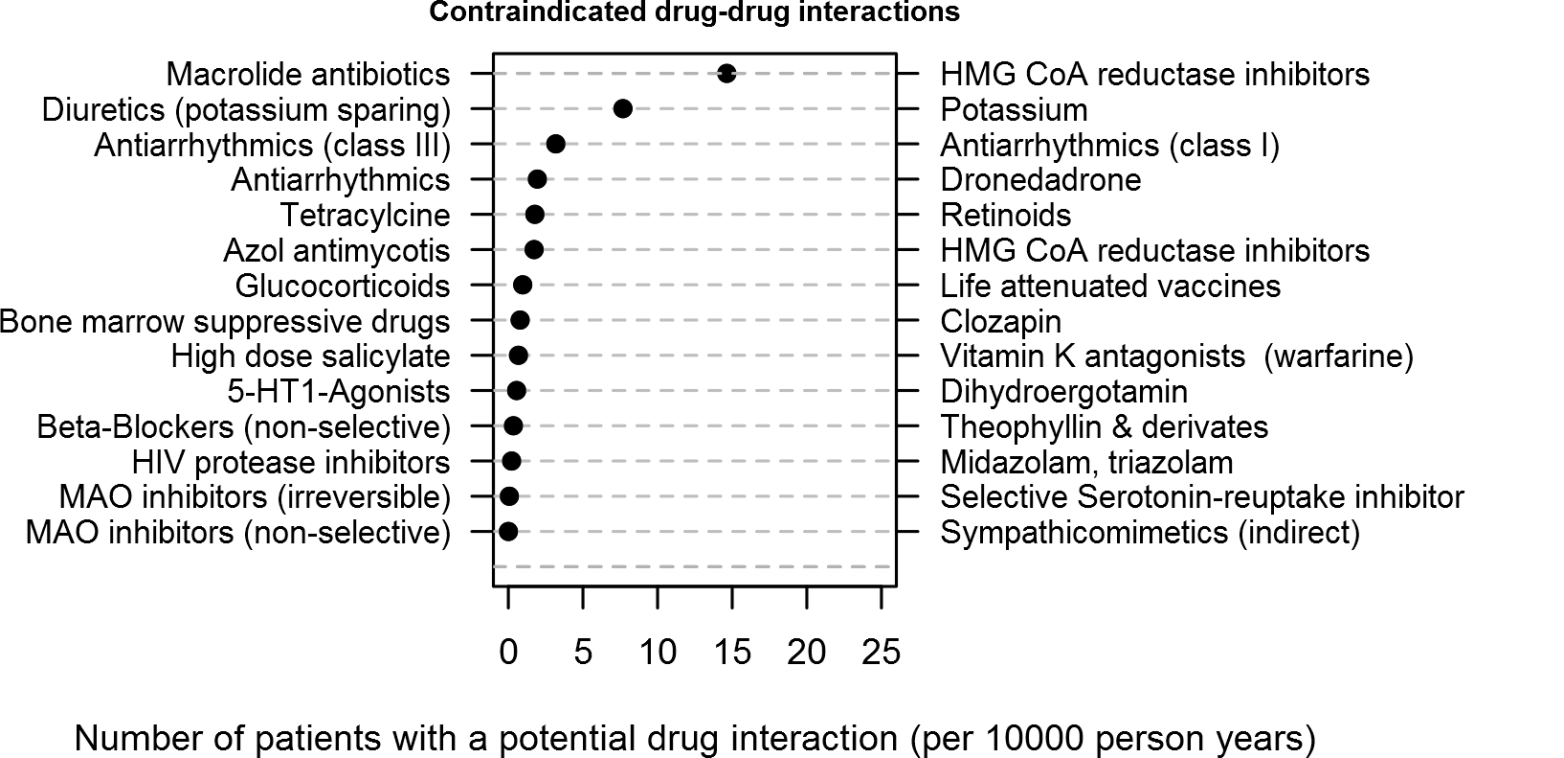
Number of patients with contraindicated drug-drug interactions Number of patients with a potential drug interaction (per 10000 person years).

The delivery date of interacting drugs occurred for 28.7% of events on the same day, and in 78.9% within 30 days. Overall the median number of patients at risk (*Ω*_*P*_*)* encompassed 30% [IQR 26%-46%] of the total number of patients of a physician. Most common contraindicated DDI (expressed per 10,000 person years) were prescriptions of macrolide antibiotics and HMG CoA reductase inhibitors, potassium sparing diuretics and potassium salt, and antiarrhythmic drug combinations of class I and class III (Fig 1).

**Fig 2:**
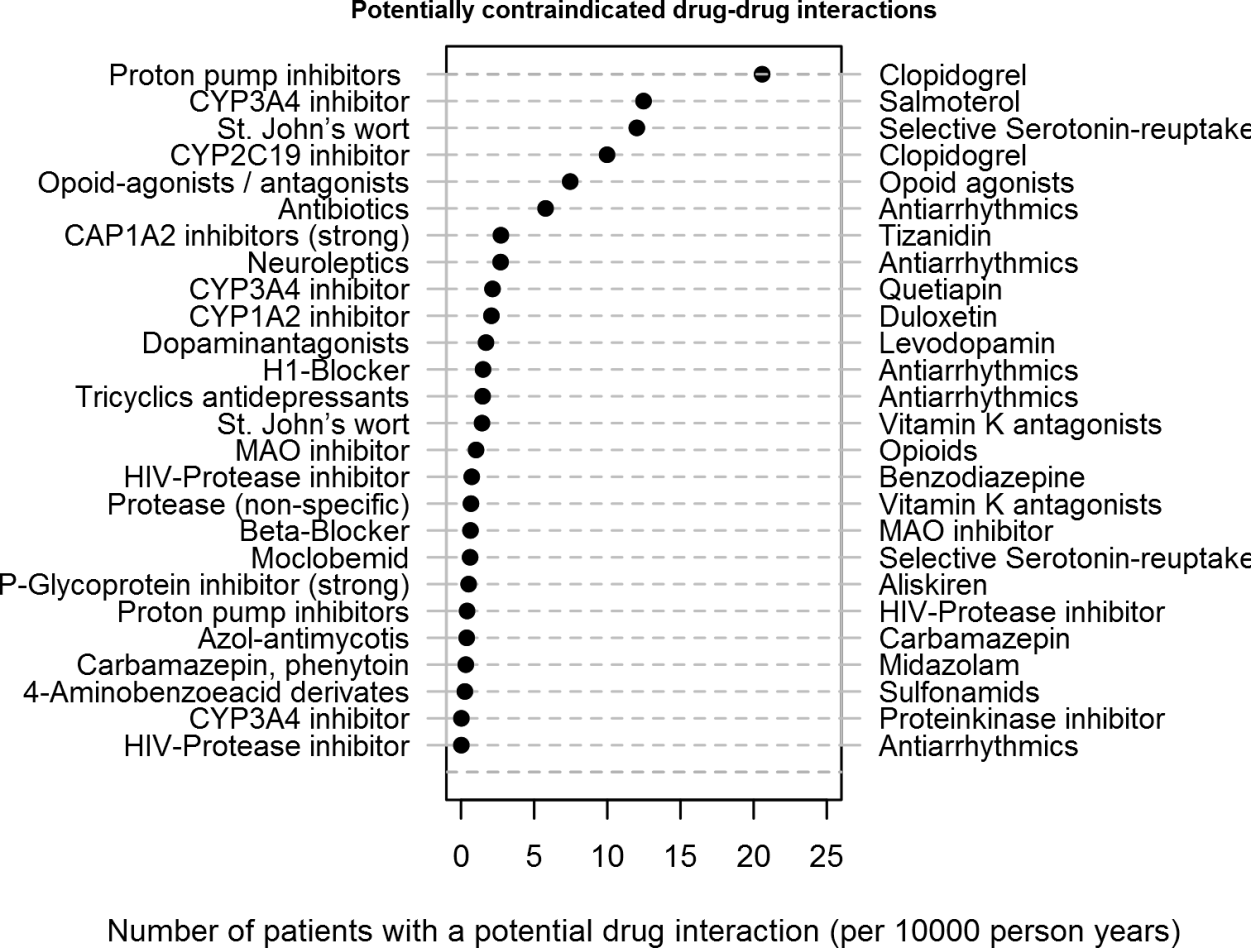
Number of patients with potentially contraindicated drug-drug interactions Number of patients with a potential drug interaction (per 10000 person years).

Most common potentially contraindicated DDIs were proton pump inhibitors with clopidogrel, cytochrome 3A4 inhibitors (such as protease inhibitors, azole antimycotics, erythromycin, clarithromycin or nefazodone) with salmeterol, and St. John's wort and serotonin reuptake inhibitors (Fig 2).

**Fig 3:**
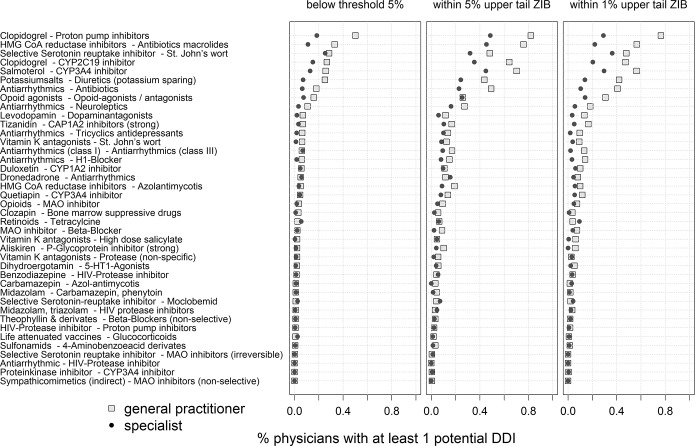
Percentage of general practitioners and specialists with at least one potential drug-drug interaction (DDI) % physicians with at least 1 potential DDI.

Fig 3 depicts common DDI by specialists. The most common potential DDI involved clopidogrel with proton pump inhibitors, HMG CoA reductase inhibitors (statins) with macrolide antibiotics, serotonin reuptake inhibitors and St. John's wort, clopidogrel with cytochrome P450-2C19 inhibitors, salmeterol with cytochrome P450-3A4 inhibitors, and potassium salt together with potassium saving diuretics. For each of these DDI the rate of general practitioners with drug prescriptions leading to potential DDI unlikely (p<0.05 & p ≥0.01) and very likely to be explained by chance was higher than in specialists.

## Discussion

This study investigated for the first time the crude prevalence of potential DDI in a large sample of approximately 40% of the Swiss population. The findings are consistent with results from population-based studies from Scandinavia which also reported a higher frequency of DDI in females and in older patients [9–11]. However, comparisons across studies are somewhat difficult as definitions of DDI differed across various observational investigations. In two Swedish population-wide studies the prevalence of DDI with serious clinical consequences during the course of one year was 1.4%, thus closely similar to our estimates, but the prevalence in female elderly individuals was 50% higher than in our study [9, 10]. In a population-based study in one county in Denmark the prevalence of major DDI was 1.9% [11].

The goal of this study was to investigate whether monitoring of DDI and tracking of potential DDI is possible on the individual physician level. Due to the large sample we were able to identify in each specialty group and for all clinically relevant contraindicated DDI physicians with an unusual prescription behaviour unlikely to be explained by chance. The detailed information in the database further allowed us to analyse for all patients the entire sequence of all prescribed drugs and to exactly determine which physician was responsible for causing a particular potential DDI. This may be seen as a particular strength of the study.

The odds for DDI vary by specialty because the type of drugs that may interact with other drugs may be related to the speciality of a physician and to the comorbidities of the served patient population. Social health insurers in Switzerland do not receive detailed diagnosis-related information from ambulatory care. For this reason we lacked relevant data on diagnoses and comorbidities which can only indirectly and rather rudimentary be assessed from pharmaceutical drug groups, for example from drugs used to specifically treat diabetes, specific neurological diseases, or COPD. For this reason we chose to restrict for each physician the study population to patients who were at least once prescribed a base drug or a counteracting drug from the predefined list of 40 DDI. We were also missing information on treatment indication and duration, dosing and refill. Therefore we had to approximate drug use periods with fixed assumptions of dosage and refill length using DDD. This approach has several disadvantages and does not consider different dosing in particular for children. The refill length was assigned as the typical duration for a single purchase which may not reflect shorter drug prescription periods. We could not use a more refined method to estimate drug use periods from historical purchase data because such models depend on the number of purchases and longer observation periods.[12]

Our intention was to investigate whether Swiss health insurer claim data can be used to monitor and track individual physicians’ prescription behaviour. Therefore it was not our intention to use a hierarchical logistic regression model including, patient age, gender and co-medication that would have allowed to adjust for risk factors known to be associated with DDI. From an epidemiological perspective this might be seen as a disadvantage. However, from a health system surveillance perspective our approach is sensitive because it allows for the identification of physicians with odd prescription behaviours that distinguishes them from their peers within a specialty. Given the lack of any information on diagnosis and comorbidity in Swiss claim data our approach seems sensitive. For these reasons we formed a reference population for each physician, which allowed to determine the accumulation of DDI at the physician level unlikely to be explained by chance. Our model is based on the assumption that populations at risk for a given drug interactions (*Ω*_*P*_) and being served by a given physician are comparable. This assumption might not reflect the heterogeneity of patient populations served by physicians. In addition, due to variation of odds across specialities DDI should only be compared within specialties which from a health system perspective might be seen as a disadvantage.

Our study has several additional limitations. The proportion of elderly patients in the study population was higher than in the general population. Due to the preponderance of these age groups we probably overestimated the prevalence of DDI in the general population to some degree. Patients from the south-east and French speaking part of Switzerland were underrepresented. In addition, patients with high deductibles can pay their drugs themselves and may not hand in drug prescriptions to their health insurance. However, internal analyses from health insurer prescription data indicate that less than 2–3% of prescriptions get thereby lost to the analyses, and therefore it is highly unlikely that many DDI have been missed [13]. In addition, patients with potential DDI are more likely to have annual costs above the deductible and will hand in their invoices. In this study we cannot account for over-the-counter drugs potentially leading to DDI. Furthermore, our analyses are based on the assumption that patients truly took all prescribed drugs, which is certainly not always the case. Our estimates relate to a 12 month period, however, large variations over time in prescription behaviour leading to DDI have been found by others. This might dilute our power to detect unusual prescription behaviour compared to studies with longer monitoring time periods [14].

Many of the contraindicated and potential DDI from our list may not be clinically relevant and can be justified with appropriate monitoring or dose adjustment. Claims data from Swiss health insurers do not encompass the necessary details which would allow to investigate or to verify the appropriateness of committed DDI. Likewise it is not possible to investigate from Swiss claims data whether potential DDI actually led to an adverse drug reaction with serious consequences and need for hospitalisation. At present no linkage with the hospitalisation statistics of Swiss DRG is possible. Given the expected low number of such events caused by an individual physician it is unlikely that adverse drug reactions might form a suitable parameter for judging the quality of a physicians’ prescription behaviour. Given the high prevalence of potential DDI in particular in the elderly population and incidence of hospital admission due to adverse drug events, intervention studies to lower potential DDI in the elderly population at large might appear useful from the public health and system perspective.

Evidence from randomized controlled intervention trials on the effectiveness of computerized decision support systems for the reduction of DDI in ambulatory care is very limited [15]. In a Canadian trial 107 general practitioners from Montreal were assigned to a computerized decisions support system versus a control group [16]. During the one year intervention period the number of discontinued drugs due to DDI nearly doubled in the intervention group compared to controls (165 versus 76 drug discontinuations per 1000 visits, relative risk 2.15, 95%CI 0.98–4.70). Preliminary evidence from a large pre- and post-intervention study with an automated DDI alert system for electronic prescribing indicated that providers stopped in a substantial proportion the prescription of 18 high-volume and high risk DDI medications (5.9% versus 10.9%), leading to a statistically non-significant decrease of definitive or probable adverse drug reactions as assessed with the Naranjo scoring system [17]. Estimates from a simulation study in Massachusetts, which was based on roughly 280,000 prescription alerts of over 2000 clinicians in ambulatory care using the PocketScript e-prescribing system, indicated that 402 adverse drug events during one year could have been averted if clinicians followed the recommendation of the e-alert system [18]. Of those, 49 were estimated to be serious and even life threatening events, and 3 leading to death. According to the model, 2715 and 44,350 alerts would have had to be checked to avert one serious adverse event and one death, respectively.

The number of physicians in ambulatory care in Switzerland with an unusual level of DDI would be sufficiently large to test whether the provision of an electronic alert system could reduce the number of DDI. Health insurer claims data would allow to identify individual physicians through the unique physician identification number and to contact and motivate them to participate in such a study. The obvious advantage of such a study would be the high external validity of findings. Future linkage to outcome data would allow assessing the impact of such an intervention on the prevention of serious adverse drug reactions, the most relevant patient important outcome.

## Supporting Information

S1 Table Drug interactions classified as ‚contraindicated‘ or ‚potentially contraindicated‘. Column reference drug indicates which substance group was used for the patient collective at risk.
(DOCX)
