# Supplementary material for: Surveillance of Physicians Causing Potential Drug-Drug Interactions in Ambulatory Care: A Pilot Study in Switzerland
Source: PLoS One. 2016 Jan 25;11(1):e0147606. doi: 10.1371/journal.pone.0147606 (PMC4726757; doi:10.1371/journal.pone.0147606)
Supplement: S1 Table — Column reference drug indicates which substance group was used for the patient collective at risk. (DOCX) [file pone.0147606.s001.docx]

## S1 Table Drug interactions classified as ‚contraindicated‘ or ‚potentially contraindicated‘. ‘

| ID number, pharmavista | category 1=contraindicated, 2= potentially contraindicated | drug class A | drug class B | Possible effect | Reference drug, A=substance in column 3, B=substance in column 4 |
| --- | --- | --- | --- | --- | --- |
| 35 | 1 | Vitamin K antagonists (phenprocoumon, acenocoumarol) | salicylates (large doses) | Increased risk of bleeding | A |
| 112 | 1 | Potassium | Diuretics, potassium-sparing | Risk of hyperkalaemia | B |
| 166 | 1 | Sympathomimetics, indirect | Monoamine oxidase inhibitors, non-selective | Increased sympathomimetic effect | B |
| 169 | 1 | Theophylline and derivatives | Beta blocking agents, non-cardioselective | Opposite effects, attenuation of bronchodilatory effect of theophylline | A |
| 253 | 1 | Antiarrhythmics (class I) | Antiarrhythmics (class III) | Increased risk of life-threatening arrhythmia | A+B |
| 348 | 1 | Retinoids | Tetracyclines | Increased risk of intracranial hypertension | A |
| 437 | 1 | Inhibitors of cholesterol synthesis (statins) | Macrolide antibiotics | Increased risk of musculoskeletal toxicity including rhabdomyolysis | A |
| 439 | 1 | Selective serotonin reuptake inhibitors | Monoamine oxidase inhibitors, irreversible | Increased risk of serotonin syndrome | A+B |
| 462 | 1 | Ergot alkaloids | Serotonin (5HT1) agonists | Increased risk of vasospastic reactions | A |
| 494 | 1 | Inhibitors of cholesterol synthesis (statins) | Azole antimycotics | Increased risk of musculoskeletal toxicity including rhabdomyolysis | A |
| 928 | 1 | Live attenuated vaccines | Glucocorticoids | Risk of disseminated infection | A |
| 981 | 1 | Clozapine | Drugs that suppress bone marrow | Increased risk or severity of bone marrow suppression | A |
| 1203 | 1 | Midazolam, triazolam | Protease inhibitors | Potential for prolonged and/or increased sedation and respiratory depression | B |
| 1265 | 1 | Dronedarone | Antiarrhythmics | Increased risk of ventricular arrhythmias including torsade de pointes | A+B |
| 139 | 2 | Opioids | Monoamine oxidase inhibitors | Risk of life-threatening disturbance of respiratory and circulatory function | B |
| 194 | 2 | Sulfonamides | 4-Aminobenzoic acid derivatives | Reduced antibacterial effects of sulfonamides | A |
| 433 | 2 | Pure opioid agonists | Agonist-antagonist opioids | Reduced analgesic effect of pure opioid agonists possible; withdrawal symptoms | A |
| 633 | 2 | Monoamine oxidase inhibitors | Beta blocking agents | Increased risk of hypotension, orthostasis, bradycardia, and heart failure due to excessive reduction of sympathetic activity | A |
| 660 | 2 | Vitamin K antagonists | St. John’s wort | Reduces hypoprothrombinemic effect of vitamin K antagonists | A |
| 686 | 2 | Quetiapine | Enzyme inhibitors (CYP3A4) | Increased effect of quetiapine | B |
| 690 | 2 | Benzodiazepine | Protease inhibitors | Accumulation of benzodiazepines with potentially serious and/or life-threatening events | B |
| 716 | 2 | Selective serotonin reuptake inhibitors | St. John’s wort | Increased risk of serotonin syndrome | A+B |
| 736 | 2 | Antiarrhythmics | Antibiotics | Increased risk of ventricular arrhythmias including torsade de pointes | A |
| 737 | 2 | Antiarrhythmics | Antidepressants, tricyclic, and analogues | Increased risk of ventricular arrhythmias including torsade de pointes | A+B |
| 738 | 2 | Antiarrhythmics | H1 blockers | Increased risk of ventricular arrhythmias including torsade de pointes | A |
| 740 | 2 | Antiarrhythmics | Neuroleptics | Increased risk of ventricular arrhythmias including torsade de pointes | A+B |
| 801 | 2 | Protein Kinase Inhibitors | Enzyme inducers (CYP3A4) | Reduced efficacy of protein kinase inhibitors | A |
| 862 | 2 | Carbamazepine | Azole antimycotics | Increased risk for adverse events of carbamazepine/reduced efficacy of azole antimycotics | A |
| 895 | 2 | Protease inhibitors | Proton pump inhibitors | Reduced efficacy of protease inhibitors | A |
| 896 | 2 | Antiarrhythmics | Protease inhibitors | Increased plasma concentrations of antiarrhythmics - increased risk of ventricular arrhythmias | B |
| 910 | 2 | Tizanidine | Enzyme inhibitors, strong (CYP1A2) | Increased effect of tizanidine | A |
| 934 | 2 | Duloxetine | Enzyme inhibitors (CYP1A2) | Increased effect of duloxetine | A |
| 1013 | 2 | Levodopa | Dopamine antagonists | Mutual reduction of effectiveness | A |
| 1014 | 2 | Serotonin reuptake inhibitors | Moclobemide (reversible inhibitor of Monoamine oxidase A) | Increased risk of serotonin syndrome | A+B |
| 1077 | 2 | Midazolam | Carbamazepine, phenytoin | Reduced sedative effect of midazolam | B |
| 1086 | 2 | Vitamin K antagonists | Proteases, unspecific | Increased risk of bleeding | A |
| 1162 | 2 | Clopidogrel | Proton pump inhibitors | Reduced cardioprotective effects of clopidogrel | A |
| 1177 | 2 | Salmeterol | Enzyme inhibitors (CYP3A4) | Increased risk of ventricular arrhythmias of salmeterol | A |
| 1199 | 2 | Aliskiren | P-glycoprotein inhibitors, strong | Excessive drop in blood pressure | A |
| 1248 | 2 | Clopidogrel | Enzyme inhibitors (CYP2C19) | Reduced cardioprotective effects of clopidogrel | A |

Column reference drug indicates which substance group was used for the patient collective at risk.
